# Polarity protein Crumbs homolog-3 (CRB3) regulates ectoplasmic specialization dynamics through its action on F-actin organization in Sertoli cells

**DOI:** 10.1038/srep28589

**Published:** 2016-06-30

**Authors:** Ying Gao, Wing-yee Lui, Will M. Lee, C. Yan Cheng

**Affiliations:** 1 The Mary M. Wohlford Laboratory for Male Contraceptive Research, Center for Biomedical Research, Population Council, 1230 York Ave, New York, 10065, New York, USA; 2 School of Biological Sciences, University of Hong Kong, Hong Kong, China

## Abstract

Crumbs homolog 3 (or Crumbs3, CRB3) is a polarity protein expressed by Sertoli and germ cells at the basal compartment in the seminiferous epithelium. CRB3 also expressed at the blood-testis barrier (BTB), co-localized with F-actin, TJ proteins occludin/ZO-1 and basal ES (ectoplasmic specialization) proteins N-cadherin/β-catenin at stages IV-VII only. The binding partners of CRB3 in the testis were the branched actin polymerization protein Arp3, and the barbed end-capping and bundling protein Eps8, illustrating its possible role in actin organization. CRB3 knockdown (KD) by RNAi in Sertoli cells with an established tight junction (TJ)-permeability barrier perturbed the TJ-barrier via changes in the distribution of TJ- and basal ES-proteins at the cell-cell interface. These changes were the result of CRB3 KD-induced re-organization of actin microfilaments, in which actin microfilaments were truncated, and extensively branched, thereby destabilizing F-actin-based adhesion protein complexes at the BTB. Using Polyplus *in vivo*-jetPEI as a transfection medium with high efficiency for CRB3 KD in the testis, the CRB3 KD testes displayed defects in spermatid and phagosome transport, and also spermatid polarity due to a disruption of F-actin organization. In summary, CRB3 is an actin microfilament regulator, playing a pivotal role in organizing actin filament bundles at the ES.

In the mammalian testis such as in rats, the most notable cell-cell anchoring junction is the testis-specific actin-rich ectoplasmic specialization (ES) (for reviews, see refs 1, 2, 3). ES is typified by the presence of a network of actin microfilament bundles that lays between the Sertoli cell plasma membrane and the cisternae of endoplasmic reticulum (for reviews, see refs 3, 4, 5). ES is limited to the Sertoli-spermatid (step 8–19 spermatids) interface in the adluminal compartment known as the apical ES, it is also found at the Sertoli cell-cell interface near the basement membrane called the basal ES^6^,^7^. Basal ES coexists with tight junction (TJ) and gap junction (GJ), and together with the desmosome, they constitute the blood-testis barrier (BTB), which physically divides the seminiferous epithelium into the basal and the adluminal (apical) compartment (for reviews, see refs 8, 9, 10). ES is a very strong anchoring junction when compared to other junctions such as the desmosome^11^, making the BTB one of the tightest blood-tissue barriers due to the presence of actin filament bundles (for reviews, see refs 8 and 12). With the exception of the desmosome wherein adhesion proteins (e.g., desmoglein-2) use intermediate filaments for attachment, adhesion proteins of the TJ (e.g., occludin, JAM-A, claudins), GJ (e.g., connexin43) and ES (e.g., nectin 2, N-cadherin) at the BTB all utilize F-actin for attachment, similar to apical ES proteins (e.g., nectin 2, nectin 3, β1-integrin, JAM-C) (for a review, see ref. 13). During the epithelial cycle, developing preleptotene spermatocytes and spermatids (step 8–19) must be transported across the BTB and the adluminal compartment of the epithelium, respectively (for reviews, see^8^,^13^). This thus involves extensively remodeling of the actin-based cytoskeleton. In short, cell junctions at the Sertoli cell-cell and Sertoli-spermatid interface are dynamic structures in which actin microfilaments must be flexibly converted between their bundled and unbundled/branched configuration to confer junction plasticity to facilitate germ cell and other organelle (e.g., phagosome) transport. Furthermore, germ cells in particular spermatids must be properly oriented so that the maximal number of spermatids can be tightly packed into the confined environment of the seminiferous epithelium with limited space to allow the daily production of ~10, ~70 and ~400 million spermatozoa from a single adult male in mice, rats, and humans^14^,^15^, respectively. As such, it is envisioned that polarity proteins play an important role in conferring spermatid polarity to maximize sperm output.

In mammalian cells, the Crumbs (CRB) protein complex, working in concert with partitioning-defective (Par) protein complex and the Scribble protein complex, regulates the formation and maintenance of epithelial polarity during embryonic development but also in adult tissues (for reviews, see refs 16, 17, 18, 19). One of the Crumbs family members, such as the Crumbs homolog-1 (CRB1), CRB2 and CRB3, forms a complex with PALS1 (protein associated with Lin-7 1, also known as Mpp5) and PATJ (PALS1 associated tight junction protein) to create a 3-protein Crumbs polarity complex found in rodents and humans, which is also the mammalian ortholog of the *Drosophila* CRB/Stardust/DmPATJ (for reviews, see refs 17 and 19). Herein, we investigate the role of CRB3 in the testis. CRB3 was selected based on the following rationale. In mammals, CRB1 is intimately related to the function of the eye, in particular photoreceptor cell polarization and adhesion. For instance, *CRB1* deletion in mice leads to localized lesions in the retinas by 3–9 months after birth^20^. In humans, mutations of *CRB1* gene lead to a variety of retinal degenerative diseases such as Leber’s congenital amaurosis (LCA)^21^, retinitis pigmentosa type 12 (RP12)^22^ and others^23^. *CRB2* expression in adult tissues is restrictively expressed in adult brain, retina, and kidney glomerulus (mostly expressed by podocytes the mouse kidney^24^) and its function is poorly understood^25^. Furthermore, *CRB2* deletion in mice leads to embryonic fatality by day E12.5 due to defects in disrupted polarity of the epiblast cells, perturbing epithelial to mesenchymal transition (EMT) during gastrulation, impairing mesoderm and endoderm formation^26^. On the other hand, *CRB3* knockout mice die shortly after birth, usually within 10 min after delivery due to respiratory distress, as a result of proteinaceous debris accumulation in the entire lung, which also coupled with cystic kidneys and disrupted microvilli in the intestine because of defects in epithelial morphogenesis^27^. More important, CRB3 is highly expressed in the rat testis^28^. Thus, we sought to examine if CRB3 is involved in modulating actin microfilament organization at the ES, regulating spermatid polarity, transport of spermatids and phagosomes in adult rat testes.

## Materials and Methods

### Animals and antibodies

Male Sprague-Dawley rats at 20 and ~70–100 (~250–300 gm b.w.) days of age were obtained from Charles River Laboratories (Kingston, NY). The use of rats for studies reported herein was approved by the Rockefeller University Institutional Animal Care and Use Committee (IACUC) with Protocol Numbers 12506 and 15780-H. All methods and experimental protocols used for relevant studies reported herein, including the use of animals, primary Sertoli cell cultures, and pertinent *in vivo* studies including the use of recombinant DNA materials such as siRNA duplexes were carried out in accordance with the relevant guidelines, including any relevant details, and approved by the Rockefeller University Laboratory Safety and Environmental Health, the Rockefeller University Institutional Biosafety Committee (IBC), and the Rockefeller University Comparative Bioscience Center (CBC). These methods were also described in details in the sections below. Rats, including 20-day-old male pups and adult animals, were euthanized by CO_2_ asphyxiation using slow, at 20–30%/min, displacement of chamber air with compressed CO_2_ in a chamber with a built-in regulator approved by the Rockefeller University Laboratory Safety and Environmental Health. Antibodies, unless specified otherwise, were obtained commercially and listed in Table 1.

### Primary Sertoli cell cultures

Sertoli cells were isolated from testes of 20-day-old pups as described^29^. Freshly isolated cells were seeded on Matrigel (1:5 ~ 1:7, diluted in F12/DMEM; BD Biosciences, San Jose, CA)-coated culture plates (for lysate preparation or RNA isolation), coverslips (for immunofluorescence microscopy) and bicameral units (for transepithelial electrical resistance (TER) measurement; Millipore, Billerica, MA) at a density 0.5, 0.04, and 1.0 × 10^6^ cells/cm^2^, respectively. Sertoli cells were cultured in serum-free F12/DMEM (Sigma-Aldrich, St. Louis, MO) supplemented with growth factors and gentamicin in a humidified atmosphere of 95% air/5% CO_2_ (v/v) at 35 °C^29^. On day 2 after Sertoli cell isolation, cells were subjected to a brief hypotonic treatment of 2.5 min, using 20 mM Tris (pH 7.4 at 22 °C) to lyse residual germ cells^30^. Sertoli cells were then rinsed to removal cellular debris and cultured overnight (or 12 h) prior to their use for experiments. These cultures were ~98% pure, with negligible contaminations of Leydig cells, peritubular myoid cells, or germ cells using specific markers for these cells by either immunoblotting (IB) or RT-PCR as previous described^31^. Sertoli cells cultured *in vitro* under the conditions described herein were shown to establish a functional TJ-permeability barrier with ultrastructures of TJ, basal ES, gap junction, and desmosome that mimic the Sertoli cell BTB *in vivo* as earlier reported^32^,^33^. Thus, this system has been widely used by investigators to study Sertoli cell BTB function^34^,^35^,^36^,^37^,^38^.

### Knockdown (KD) of CRB3 by RNAi in Sertoli cells

Sertoli cells were cultured *in vitro* for 2 days as described above with an established functional TJ-permeability barrier, containing ultrastructures of TJ, basal ES, gap junction and desmosome^32^,^33^. These cells were then transfected with non-targeting negative control siRNA duplexes (Cat. No. 4390844, Ambion) *vs.* ON-TARGETplus SMARTpool CRB3 siRNA duplexes mixture (Cat. No. J-097399-09: 5′-AGGCCAUCAUCACGACCAA-3′; J-097399-10: 5′-CACAAAUAGCACAACUCAA-3′; J-097399-11: 5′-GAUAGGUACAAUAAAGGUU-3′; J-097399-12: 5′-CUGGUGGGCUAUACAGCAU-3′, Dharmacon, GE Healthcare, Lafayette, CO). Furthermore, two non-functional CRB3 siRNA duplexes (Cat. No. 4390771; siRNA ID, s153670: 5′-CAGUCAGACUCAUACAAUAtt-3′ (RNAi #1); 4390771; siRNA ID,s153671: 5′-CACCAGACUCUUUCACAAAtt (RNAi#2), Ambion/Life Technologies/ThermoFisher) identified in pilot experiments and were used for transfection in some selected subsequent experiments to serve as internal negative controls. All siRNA duplexes were used at 150 nM with Lipofectamine RNAiMAX reagent (Life Technologies, Carlsbad, CA) as the transfection medium and knockdown was performed for 24 h. Thereafter, cells were rinsed with F12/DMEM twice and then cultured in F12/DMEM supplemented with growth factors for 12 h to allow recovery. Based on results of pilot experiments, cells needed to be transfected with siRNA duplexes for another 24 h (second transfection) in order to silence CRB3 by ~50% to yield reproducible phenotypes. About 12 h after the second transfection, cells were harvested for lysate preparation, RNA isolation, actin bundling assay, or subjected to TER measurement. For immunofluorescence staining, Sertoli cells were co-transfected with 100 nM siRNA duplexes and 1 nM siGLO Red Transfection Indicator (Dharmacon) on day 3. After 24 h, cells were rinsed twice and cultured with fresh F12/DMEM including supplements for an additional 24 h before termination to be used for IF analysis.

### Knockdown of CRB3 in adult rat testes *in vivo*

To knockdown CRB3 *in vivo*, 8 adult rats (~270–300 g body weight) were transfected with non-targeting control *vs.* CRB3 siRNA duplexes via intratesticular injection using a 28-gauge needle as described^39^. It is noted that this regimen was established based on results of two pilot experiments. On day 0, one testis of a rat received non-targeting control siRNA, the other testis received CRB3 siRNA using Polyplus *in vivo*-jetPEI^®^ (Polyplus-transfection S.A., Illkirch, France) as a transfection medium. In brief, siRNA duplexes (150 nM) were constituted in 100 μl of transfection solution containing ~0.4 μl *in vivo*-jetPEI^®^ according to the manufacturer’s instruction using an N/P ratio of 8 (note: N/P ratio is a measure of the ionic balance of the siRNA complexes, referring to the number of nitrogen residues of jetPEI^®^/oligonucleotide phosphate, in which jetPEI^®^ concentration is expressed in nitrogen residues molarity and 1 μg of oligonucleotide contains 3 nmol of anionic phosphate). This transfection solution in 100 μl was administered to each testis (~1.6 g in weight with a volume of ~1.6 ml) using a 28-gauge needle attached to a 0.5 ml insulin syringe by gently inserting from the apical to near the basal end of the testis vertically. As the needle was withdrawn apically, transfection solution was released gently and gradually from the syringe so that the entire testis was slowly filled with the transfection solution that spreaded across the entire testis to avoid an acute rise in intratesticular hydrostatic pressure. Rats were euthanized by CO_2_ asphyxiation on day 5. Testes from 5 rats were snap-frozen in liquid nitrogen, and stored at −80 °C until used for immunoblotting, IF and/or qPCR. Testes from 3 rats were fixed in Bouin’s fixative to obtain paraffin sections for histological analysis using hematoxylin and eosin (H&E) staining.

### Lysate preparation and immunoblotting (IB)

Protein lysates from primary Sertoli cells or testes were prepared in IP lysis buffer [50 mM Tris, pH 7.4 at 22 °C, containing 0.15 M NaCl, 1% NP-40 (v/v), 2 mM EGTA, and 10% glycerol (v/v),] supplemented with protease and phosphatase inhibitors (1 mM 4-(2-aminoethyl)benzene sulfonyl fluoride hydrochloride, 1 mM sodium orthovanadate, 0.05 mM bestatin, 0.05 mM sodium EDTA, 15 μM E64, 1 mM pepstatin, 4 mM sodium tartrate dehydrate, 5 mM NaF, and 3 mM β-glycerophosphate disodium salt). Protein concentration was determined using a DC Protein Assay kit from Bio-Rad (Hercules, CA). Equal amount of lysates between samples (~30–100 μg protein) were resolved by SDS-PAGE and transferred onto nitrocellulose membrane (Bio-Rad) for immunoblotting. Membranes were blocked with 5% non-fat milk powder (w/v) in PBS (10 mM sodium phosphate, 0.15 M NaCl)/Tris (10 mM)/Tween-20 (0.1%, v/v), pH 7.4 at 22 °C, for 1 h, to be followed by an incubation with primary antibody at 4 °C overnight. Membranes were then incubated with the corresponding secondary antibody (Santa Cruz biotechnology, Santa Cruz, CA) for 1 h. Chemiluminescence was performed using a kit performed in-house as described^40^. Images on blots were captured using a Fujifilm LAS-4000 mini-Luminiscent Image Analyzer and images were acquired and analyzed using the Fujifilm Multi-Gauge software package (Version 3.1).

### Immunohistochemistry (IHC), dual-labeled immunofluorescence (IF) and histological analysis

IHC analysis was performed using frozen section of testes (7 μm thickness) obtained in a cryostat at −22 °C. Thereafter, sections were fixed in 4% paraformaldehyde (PFA) in PBS for 10 min. Sections were blocked with 10% normal goat serum (v/v, in PBS) and then incubated with CRB3 antibody (Table 1) at 4 °C overnight. Thereafter, sections were incubated with biotinylated secondary antibody, followed by an incubation with streptavidin-horseradish peroxidase (Life Technologies). Color development was performed using aminoethyl carbazole (AEC) kits from Life Technologies. Dual-labeled IF was performed using frozen section of testes (7 μm thickness) or Sertoli cells cultured on coverslips at a density of 0.04 × 10^6 ^cells/cm^2^. Sections and/or cells were fixed in 4% PFA, and permeabilized in 0.1% (v/v) Triton X-100 in PBS or 1% SDS (w/v) in PBS, and subsequently blocked in 10% normal goat serum (v/v) or 1% BSA (w/v) in PBS. Sections and/or cells were then incubated with primary antibodies (Table 1) at 4 °C overnight, followed by Alexa Fluor 488 (green) or Alexa Fluor 555 (red)-conjugated secondary antibodies (Life Technologies) for 1 h. An anti-CRB3 antiserum used to visualize CRB3 in Sertoli cells by immunofluorescence was a kind gift from Dr. Ben Margolis which was earlier characterized^41^, wherein Sertoli cells were seeded on coverslips at a density of 0.15 × 10^6 ^cells/cm^2^ and immunofluorescence staining was performed as described^41^. To visualize F-actin, sections and/or cells were incubated with fluorescein isothiocyanate (FITC)-conjugated phalloidin (Life Technologies) at 1:50 dilution for 1 h. Slides were mounted in Prolong Gold Antifade reagent with 4′, 6-diamidino-2-phenylindole (DAPI, Life Technologies). Histological analysis was performed using testes fixed in Bouin’s fixative to obtain paraffin sections and stained for hematoxylin and eosin (H&E). Fluorescence, IHC and H&E stained images were obtained using a Nikon Eclipse 90i Fluorescence Microscope equipped with a Nikon Ds-Qi1Mc digital camera, using Nikon NIS Elements Imaging software package (Nikon Instruments, Inc). Image files were analyzed using Photoshop in Adobe Creative Suite (Version 3.0; San Jose, CA) for image overlay to assess protein co-localization. All sections of testes or Sertoli cells within an experimental group were processed simultaneously in a single experimental session to eliminate inter-experimental variations. All experiments were repeated at least 3 times using different preparation of cells or testes from *n* = 3 rats including both control and treatment groups.

### Image analysis

For *in vitro* silencing in Sertoli cells, at least 200 cells were randomly selected and examined in control *vs.* experimental groups with *n* = 3 experiments (i.e., ~70 randomly selected cells per experiment) in which either the fluorescence signals or changes in the distribution of a target protein at the cell-cell interface were scored. For changes in protein localization near the Sertoli cell surface, the distribution of fluorescence signals at the cell-cell interface was measured at the two opposite ends (i.e., 4 measurements) of the Sertoli cell nucleus, which was then averaged to obtain the mean width. Fluorescence intensity of a target protein in Sertoli cells or in the seminiferous epithelium of testes following *in vivo* silencing was quantified using ImageJ 1.45 software (NIH, Bethesda, MD; http://rsbweb.nih.gov/ij). At least 80 randomly selected stage VIII *vs*. stage IX tubules from cross-sections of a rat testis were examined with *n* = 5 rats. Analysis was focused on these two stages because defects in spermatid transport that impeded spermiation, loss of spermatid polarity, and defects in phagosome transport were readily detected in these stages.

### Co-immunoprecipitation (Co-IP)

Co-IP was performed as described^28^. In brief, about 800 μg protein lysates from adult rat testes or 400 μg protein lysates from Sertoli cells (cultured alone for 4 days at 0.4 × 10^6 ^cells/cm^2^ on Matrigel-coated dishes) was diluted in IP lysis buffer and incubated with 1.5 μg normal rabbit or mouse IgG for 2 h, to be followed by 2 h of incubation with 15 μl Protein A/G PLUS agarose (Santa Cruz) to precipitate proteins that interacted non-specifically with IgG and/or Protein A/G PLUS agarose. This pre-cleared lysate was then incubated with 2 μg primary immunoprecipitating antibody, such as anti-CRB3 *vs.* others (see Table 1), at room temperature overnight. Protein A/G PLUS agarose (20 μl) was added and incubated at room temperature for 6 h to recover the immunocomplexes by centrifugation (1000 *g*, 5 min at 4 °C), which were then washed with IP lysis buffer (4X, 1000 g, 5 min at 4 °C each, by resuspending pellet by agitation gently), and proteins in the immunocomplexes were then extracted in SDS sample buffer and used for immunoblotting to identify interacting partner proteins. In selected experiments, the efficiency of Co-IP was assessed as follows. ~800 μg testis lysate was precipitated by corresponding antibodies (2 μg) *vs.* normal IgG of the same species that served as a control. Protein A/G PLUS agarose was used to pull down the immunocomplexes. After immunoprecipitation, 40 μl supernatant from 250 μl sample from both groups was analyzed by immunoblotting to assess if there was a considerably loss of a target protein from the sample immunoprecipitated with a primary antibody against the target protein *vs.* control (normal IgG of the same species). All Co-IP experiment was performed with *n* = 3 independent experiments which yielded similar results.

### Assessment of Sertoli cell TJ-permeability *in vitro*

Sertoli cells were plated on Matrigel (1:5)-coated bicameral units (diameter, 12 mm; pore size, 0.45 μm; effective surface area, 0.6 cm^2^) at 1 × 10^6 ^cells/cm^2^. Bicameral units were then placed in 24-well plates with 0.5 ml F12/DMEM each in the apical and basal compartment. To assess Sertoli cell TJ-permeability barrier, both the silencing and the control group had triplicate bicameral units and TER (transepithelial electrical resistance) across the cell epithelium was recorded daily. It is noted that TER monitored the ability of the Sertoli cell epithelium (i.e., the TJ-barrier) to resist conductivity of electrical current that was sent across the electrodes of the Millipore Millicell ERS system as described^29^. Each experiment was repeated at least 3 times using different batches of Sertoli cells which yielded similar results.

### Actin bundling assay

Actin bundling assay was performed using an Actin Binding Protein Spin-Down Assay Biochem Kit from Cytoskeleton (Cat No. BK013, Denver, CO) as earlier described^39^,^42^. In brief, Sertoli cell lysates obtained from cells in treatment *vs.* control groups were prepared in Tris lysis buffer [20 mM Tris, pH 7.5 at 22 °C, containing 20 mM NaCl and 0.5% Triton X-100, freshly supplemented with protease and phosphatase inhibitors] as described^42^. Cell debris was removed by centrifugation at 20,817 *g* for 1 h at 4 °C. Protein concentration was determined using a DC Protein Assay kit (Bio-Rad). Purified non-muscle actin was reconstituted to 10 mg/ml at 4 °C with sterile water. Actin was first diluted to 1 mg/ml with general actin buffer [5 mM Tris-HCl, pH 8.0 at 22 °C containing 0.2 mM CaCl_2_ and freshly supplemented with 0.2 mM ATP]. Diluted actin was then incubated on ice for 30 min to obtain G-actin. Thereafter, 10× actin polymerization buffer [100 mM Tris-HCl pH 7.5 at 22 °C containing 500 mM KCl, 20 mM MgCl_2_ and 10 mM ATP] was added and incubated at room temperature for 1 h to allow the assembly of F-actin from G-actin. Cell lysate or 10 μl Tris lysis buffer (served as a negative control) was added to the F-actin prepared above, and incubated at room temperature for 1 h to allow actin bundling. This mixture was centrifuged at 14,000 *g* for 5 min at 24 °C to sediment bundled F-actin in the pellet, whereas the linear and unbundled actin microfilaments remained in the supernatant. The whole pellet resuspended in 30 μl sterile water from each sample, and an aliquot of 5 μl supernatant from each sample were used for analysis by immunoblotting. All samples within an experimental group vs. controls, including negative control, were performed and analyzed simultaneously to avoid inter-experimental variations. This experiment was repeated 4 times using different cell preparations and yielded similar results.

### RNA isolation, RT-PCR and real-time PCR (qPCR)

Total RNA was isolated from testis, Sertoli and germ cells using Trizol reagent (Life Technologies) and reverse transcribed with Moloney murine leukemia virus reverse transcriptase (Promega, Madison, WI) as described^43^. RT products were used as templates for subsequent PCR with a primer pair specific for a target gene (e.g., CRB3) *vs.* S16 primers (Table 2). Co-amplifications of a target gene and S16 were in their linear phases. The authenticity of the PCR product was confirmed by direct gene sequencing at Genewiz (South Plainfield, NJ). For qPCR, the mRNA level of CRB3 was analyzed by QuantStudio^TM^ 12K Flex Real-Time PCR System (Thermo Fisher, Waltham, MA) with Power SYBR Green Master Mix (Applied Biosystems, Foster City, CA) according to the manufacturer’s instructions (*n* = 5, each in triplicates). GAPDH was used as an internal control for normalization. The specificity of the fluorescent signal was verified by both melting curve analysis and gel electrophoresis. The expression level of the target gene was determined using 2^−ΔΔC^_T_ method.

### Statistical analysis

For studies using Sertoli cell cultures, triplicate coverslips, dishes, or bicameral units were used for each experiment including treatment and control groups. Each data point (or bar graph) is a mean ± SD of *n* = 3 to 5 experiments (or *n* = 5 rats). Statistical analysis was performed using the GB-STAT software package (version 7.0; Dynamic Microsystems, Silver Spring, MD). Statistical analysis was performed by two-way ANOVA followed by Dunnett’s test. In selected experiments, Student’s *t-*test was used for paired comparisons.

## Results

### Expression of CRB3 and its component proteins in adult rat testes

**Figure 1:**
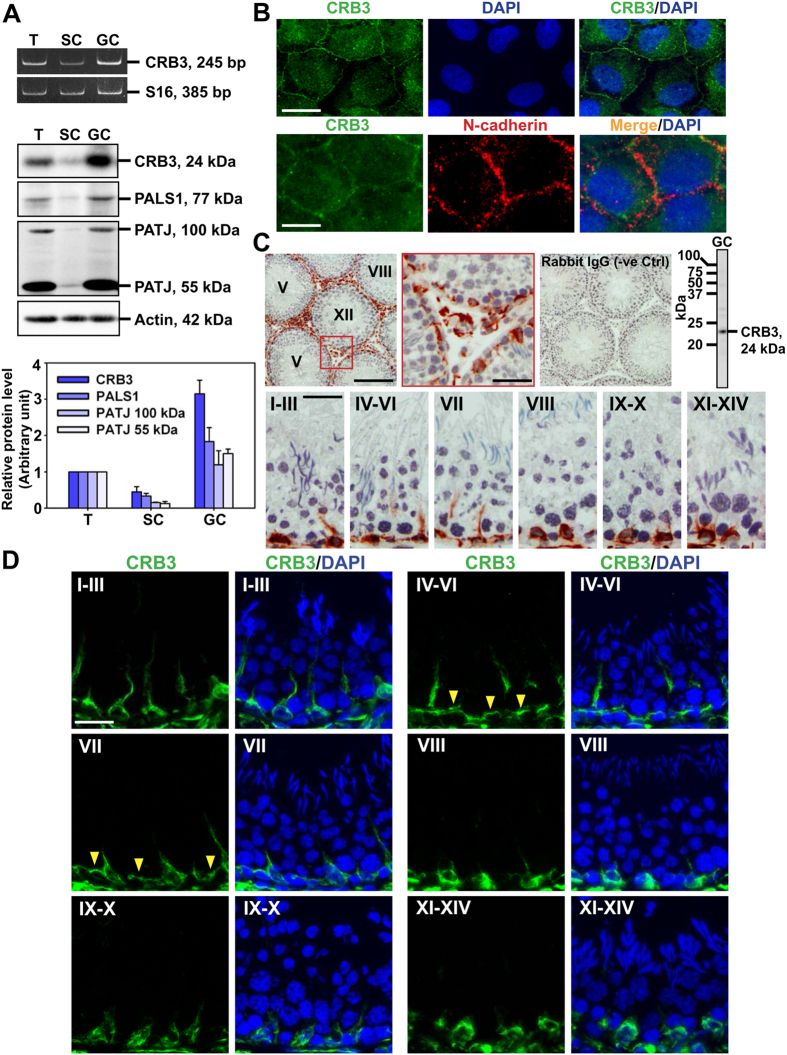
Characterization of polarity protein CRB3 in the rat testis. (**A**) Steady-state mRNA (upper panel) and protein (lower panel) levels of CRB3 and its partner proteins PALS1 and PATJ that constitute the CRB3-based polarity protein complex in adult rat testes (T) *vs.* Sertoli cells (SC), and total germ cells (GC). RT-PCR was performed using a CRB3 primer pair (Table 2) with S16 served as a loading control. For immunoblotting, β-actin served as a loading control. (**B**) Localization of CRB3 in Sertoli cells cultured for 4 days using with an anti-CRB3 antiserum (green fluorescence) (Table 1), either alone (upper panel) or co-localized with N-cadherin (red fluorescence) (lower panel). Cell nuclei were stained by DAPI (blue). Scale bar, 20 μm for upper panel; 10 μm for lower panel. (**C**) Localization of CRB3 in the seminiferous epithelium at stages I-XIV of the epithelial cycle by IHC using frozen sections of adult rat testes, illustrating CRB3 was localized mostly at the basal compartment, near the tunica propria and associated with Sertoli cells (see the reddish-brown stalks), undifferentiated spermatogonia and early spermatocytes prior to their differentiation into pachytene spermatocytes (see lower panel). Boxed area in red in the upper panel was magnified, illustrating CRB3 also expressed in Leydig cells, and endothelial cells of the microvessels, in the interstitium. Negative control (−ve Ctrl) was performed in which the primary antibody was substituted by normal rabbit IgG and with the corresponding secondary antibody, illustrating the staining was specific for CRB3. Antibody specificity was also assessed by immunoblotting using germ cell lysates that yielded a prominent band corresponding to the Mr of CRB3 at 24 kDa. Scale bars, 250 μm, and 80 μm in inset, for upper panel; 40 μm for lower panel. (**D**) Stage-specific expression of CRB3 (green fluorescence) in the seminiferous epithelium of adult rat testes, illustrating CRB3 was restrictively expressed in the basal compartment near the tunica propria, consistent with data of IHC shown in (**C**). CRB3 apparently expressed at the BTB but limited to stages IV-VI and VII tubules (see yellow arrowheads). Cell nuclei were visualized by DAPI (blue). Scale bar, 30 μm.

CRB3 was expressed by Sertoli and germ cells in the testis as demonstrated by RT-PCR (Fig. 1A) using a primer pair specific to CRB3 with S16 as the loading control (Table 2). The PCR product was verified to be CRB3 by direct nucleotide sequence analysis. CRB3 and its two component proteins PALS1 and PATJ, similar to *Drosophila* and other species, were also expressed in Sertoli and germ cells *vs.* adult testes as illustrated by immunoblotting using the corresponding specific antibodies (Fig. 1B and Table 1). These findings also suggest that germ cells express relatively more CRB3 complex *vs.* Sertoli cells (Fig. 1A, lower panel). When Sertoli cells were cultured *in vitro* for 4 days with an established functional TJ-barrier, containing ultrastructures of TJ, basal ES, GJ and desmosome that mimicked the BTB *in vivo* as earlier reported^33^,^44^, CRB3 was mostly localized at the cell-cell interface (Fig. 1B) using an anti-CRB3 antibody (Table 1). This pattern of cellular distribution of CRB3 in Sertoli cells is consistent with findings using other mammalian cells and the fact that CRB3 is an integral membrane protein^45^,^46^. CRB3 also co-localized with N-cadherin, a basal ES integral membrane protein at the BTB (for a review, see ref. 13), at the Sertoli cell-cell interface (Fig. 1B). Specificity of the CRB3 antibody was supported in a study by immunoblotting using lysates of germ cells since only a prominent band corresponding to the apparent molecular weight (Mr) of CRB3 of 24 kDa was detected (Fig. 1C). This anti-CRB3 antibody was then used for IHC (Fig. 1C) and immunofluorescence analysis (IF) (Fig. 1D). CRB3 was localized almost exclusively to the basal compartment of the seminiferous epithelium, nearing the basement membrane as noted by IHC (Fig. 1C) and IF (Fig. 1D), and also appeared as projections (or stalks) along the cell-cell interface between Sertoli cells in stages I-VII but not at stage VIII and thereafter (Fig. 1C,D). CRB3 appeared to wrap around Sertoli cells, undifferentiated spermatogonia and early spermatocytes, such as preleptotene spermatocytes, but not in pachytene spermatocytes and more advanced spermatocytes and haploid spermatids (Fig. 1C,D). CRB3 was also detected in Leydig cells and endothelial cells of the microvessels in interstitium (Fig. 1C).

### CRB3 is expressed at the basal ES/BTB stage specifically

**Figure 2:**
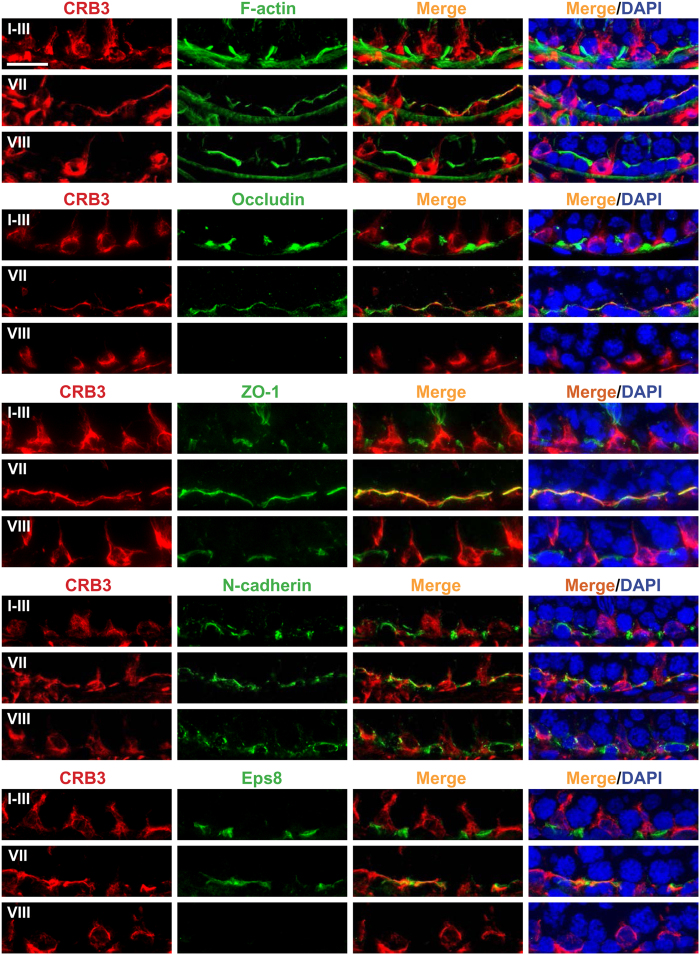
Stage-specific expression of CRB3 at the Sertoli cell BTB in adult rat testes. Dual-labeled immunofluorescence analysis illustrating co-localization of CRB3 (red fluorescence) with actin microfilaments (F-actin, green fluorescence), TJ proteins occludin and ZO-1 (green fluorescence), basal ES protein N-cadherin (green fluorescence) and actin bard-end capping and bundling protein Eps8 (green fluorescence) at the BTB. However, CRB3 expression at the BTB restricted to stage VII tubules (and also stages IV-VI tubules, see Fig. 1D) since CRB3 expression was not detected at the BTB and failed to co-localize with F-actin, occludin, ZO-1, N-cadherin and Eps8 in stages I-III and VIII tubules. Instead, at stages I-III and VIII, CRB3 was localized to Sertoli cells (e.g., Sertoli cell stalk), undifferentiated spermatogonia and early spermatocytes prior reaching the pachytene stage. Cell nuclei were visualized by DAPI (blue). Scale bar, 30 μm.

Results of IF suggested that CRB3 was expressed at the BTB but only at stages IV-VII, see yellow arrowheads in Fig. 1D, but not at stages VIII-XIV (Fig. 1D). To further characterize the stage specific expression of CRB3 at the BTB, double labeled immunofluorescence analysis was perform to assess co-localization of CRB3 with F-actin and several putative BTB-associated proteins, such as occludin (a TJ integral membrane protein), ZO-1 (a TJ adaptor protein), N-cadherin (a basal ES integral membrane protein), and Eps8 (a TJ actin barbed end capping and bundling protein) in the seminiferous epithelium of adult rat testes in stage VII *vs.* I-III and VIII tubules (Fig. 2). Consistent with findings shown in Fig. 1D, CRB3 was found to co-localize with F-actin and the other four putative basal ES/BTB proteins at stage VII only, but not in I-III and VIII (Fig. 2), illustrating its stage specific expression at the BTB. It was also noted that at stage VIII, the expression of occludin and Eps8 was considerably diminished when BTB underwent remodeling to facilitate the transport of preleptotene spermatocytes at the site, consistent with findings of earlier reports^47^,^48^.

### PATJ, a component protein of the CRB3-based polarity protein complex, is expressed at the basal ES/BTB and apical ES stage-specifically during the epithelial cycle

**Figure 3:**
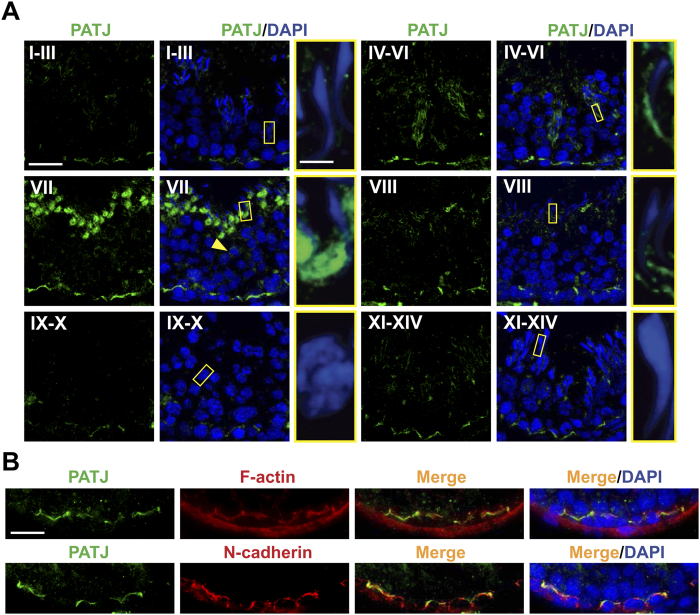
Stage-specific expression of the CRB3-associated protein PATJ in the seminiferous epithelium of adult rat testes. (**A**) Localization of PATJ (green fluorescence) in the seminiferous epithelium at stages I-XIV using frozen sections of adult rat testes for IF, illustrating that PATJ was expressed at the BTB at all stages but the highest in stages IV-VII. In addition, this expression pattern is similar to CRB3 shown in Fig. 1D. Unlike CRB3, PATJ was also detected at the apical ES at the interface of elongating/elongated spermatids and Sertoli cells from stages IV-VIII. At stages IV-VI, PATJ was expressed at the convex side of spermatids, but its expression shifted to the concave side of spermatids at stage VII, and at stage VIII, the expression of PATJ was considerably diminished and it also shifted to the tip of spermatid heads. Some weak PATJ expression was also detected in round spermatids (yellow arrow). Boxed area in yellow was magnified and shown in inset. Cell nuclei were visualized by DAPI (blue). Scale bar, 30 μm; scale bar in inset, 5 μm; which apply to other micrographs. (**B**) Dual-labeled IF analysis illustrating co-localization of PATJ (green fluorescence) with F-actin (red fluorescence) and basal ES protein N-cadherin (red fluorescence) at the BTB in stage VII tubules. Cell nuclei were visualized by DAPI (blue). Scale bar, 30 μm.

Since PALS1 and PATJ are known binding partners of CRB3 (for reviews, see refs 19 and 49), we attempted to localize PALS1 and PATJ in the testis by IF using frozen sections of rat testes. However, several antibodies against PALS1 obtained commercially failed to yield satisfactory results, but an anti-PATJ antibody was available and generated consistent staining pattern (see Table 1). Similar to CRB3, PATJ was expressed at the BTB at all stages of the epithelial cycle, and with the highest expression from stages IV-VII, consistent with CRB3 stage-specific expression at the BTB (Fig. 3A *vs.* Fig. 1C,D). Additionally, PATJ was also localized at the apical ES at the elongating/elongated spermatid-Sertoli cell interface from stages IV-VIII, but most prominently at stage VII (Fig. 3A). At stages IV-VI, PATJ was localized at the convex side of spermatid, but shifted to the concave side of elongated heads at stage VII, and then it moved to the tip of spermatid heads at stage VIII (Fig. 3A), consistent with the observation that PATJ is also a binding protein of Par3 and Par6 which are components of the apical ES^28^. Some weak PATJ staining was also detected in round spermatids (Fig. 3A). Collectively, these findings are consistent with previous studies showing that PATJ is localized at the apical ES and round spermatids in mouse^50^ and rat testes^28^. Furthermore, PATJ co-localized with F-actin and basal ES protein N-cadherin at the BTB, such as at stage VII of the epithelial cycle (Fig. 3B).

### CRB3 structurally interacts with PALS1, PATJ, actin binding/regulatory proteins Arp3, Eps8 and basal ES protein N-cadherin

**Figure 4:**
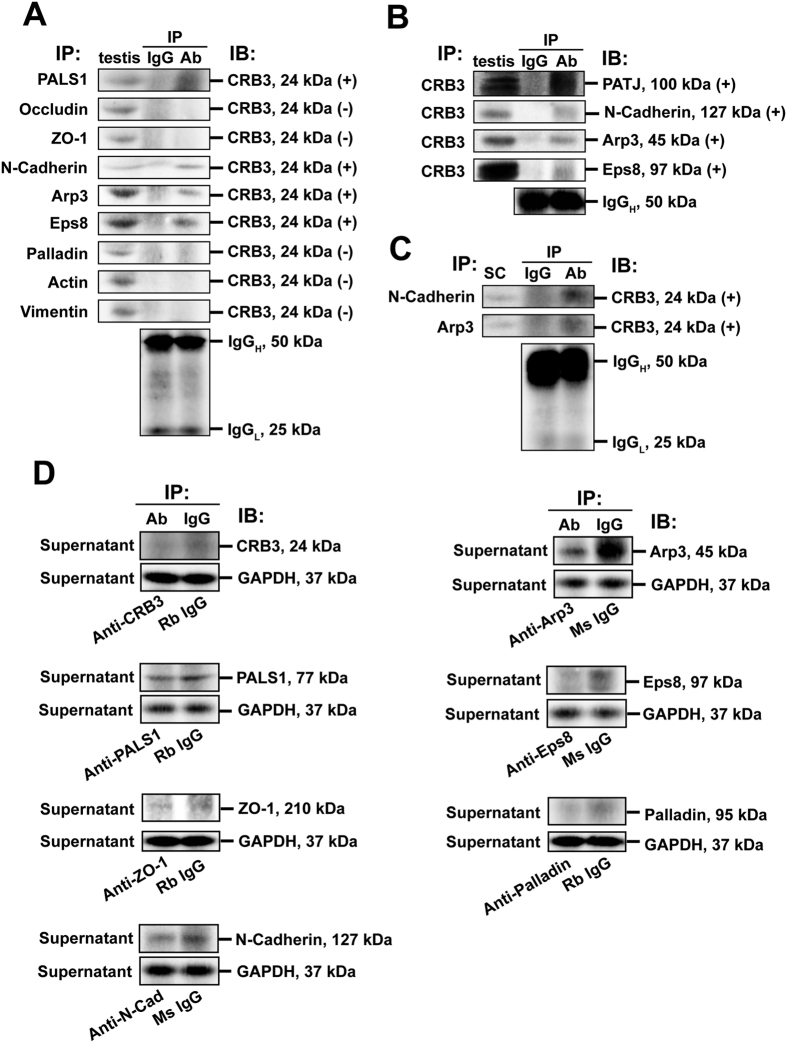
Characterization of the binding partners of CRB3 at the BTB by co-immunoprecipitation (Co-IP). (**A**) A study by Co-IP to assess protein-protein interactions between CRB3 and selected BTB-associated proteins, such as CRB3-associated protein (e.g., PALS1), TJ proteins (e.g., occludin, ZO-1), basal ES protein (e.g., N-cadherin), actin binding/regulatory proteins (e.g., Arp3, Eps8, palladin), and cytoskeletal proteins (e.g., actin, vimentin) at the BTB using corresponding primary antibodies (Table 1) and lysates from adult rat testes for immunoprecipitation. Rabbit or mouse IgG served as a negative control for the Co-IP experiment, whereas normal testis lysate without Co-IP served as a positive control. The heavy and light chains of IgG served as loading control. (+), positive interaction; (−), negative (or no) interaction. (**B**) Results of a Co-IP experiment using CRB3 antibody as the immunoprecipitating antibody to confirm interactions between CRB3 and its binding partners shown in (**A**). Normal rabbit IgG was used for Co-IP and served as a negative control, whereas normal testis lysate without Co-IP served as a positive control. The heavy chain of IgG served as a loading control. (**C**) Results of Co-IP to confirm interactions between N-cadherin and Arp3 with CRB3 using lysates from Sertoli cells. (**D**) A study to confirm the efficacy of the Co-IP in which a primary antibody was assessed for its ability to pull out itself *vs.* normal IgG of the same species which served as the corresponding control. It was noted that the primary antibodies used for Co-IP efficiently pulled down the corresponding target proteins since these proteins were considerably reduced in the supernatant when compared with the control immunoprecipitated with normal IgG of the same species. GAPDH in the supernatant confirmed equal protein loading between samples for Co-IP. Rb, rabbit; Ms, mouse; N-Cad, N-cadherin. Data shown herein are representative findings of a representative experiment from *n* = 3 independent experiments with similar results.

Crumbs (CRB) forms a complex with PALS1 and PATJ to create the Crumbs-based polarity complex to confer cell polarity (for reviews, see refs 19 and 49). Herein, a study by Co-IP indeed confirmed CRB3 interacted structurally with PALS1 and PATJ in rat testes (Fig. 4A,B). Due to its stage-specific localization in IV-VII tubules at the basal ES/BTB (Figs 1C,D and 2), which is an actin-rich cell adhesion ultrastructure, we next examined if CRB3 structurally interacted with BTB-associated proteins in particular actin binding proteins (ABPs) by Co-IP (Fig. 4A,B). Indeed, CRB3 was found to interact with two ABPs Arp3 (a barbed end nucleation protein that effectively confers F-actin to organize as a branched network) and Eps8 (a barded end capping and bundling protein) which are known to induce remodeling of actin microfilaments in mammalian cells, effectively converting actin microfilaments from their bundled to unbundled/branched configuration and vice versa at the Sertoli cell BTB^51^, and also basal ES protein N-cadherin (Fig. 4A,B). To further confirm specific interaction between CRB3 and ABPs, CRB3 was found to interact with N-cadherin and Arp3 using lysates of Sertoli cells (Fig. 4C). Efficiency of Co-IP was also confirmed as noted in Fig. 4D since the presence of immunoprecipitating antibody of a target protein (e.g., CRB3, PALS1, ZO-1, N-cadherin, Arp3, Eps8 and palladin) considerably reduced the level of the target protein in the supernatant of the Co-IP experiments vs. the corresponding controls using normal IgG of the same animal species with GAPDH served as the loading control (Fig. 4D).

### CRB3 knockdown (KD) in Sertoli cells with an established functional TJ-permeability barrier perturbs TJ barrier function via changes in the distribution of BTB-associated proteins

**Figure 5:**
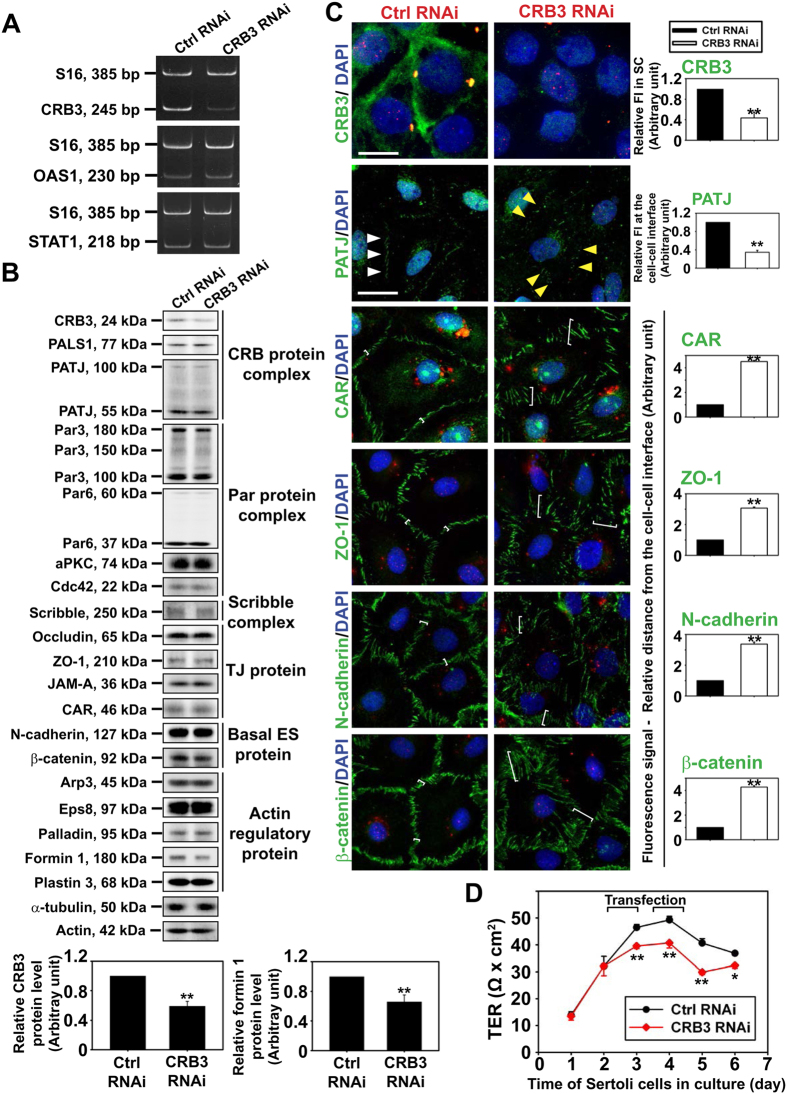
CRB3 KD perturbs the Sertoli cell TJ-permeability barrier. Sertoli cells cultured for 2 days were transfected with corresponding siRNA duplexes at 150 nM for 24 h. Cells were allowed to recover for 12 h, to be followed by a second transfection for 24 h. After 12 h, cells were harvested for PCR or IB. For IF analysis, Sertoli cells were transfected with 100 nM siRNA on day 3 for 24 h, cells were rinsed and cultured for 24 h before termination. (**A**) RT-PCR confirmed considerable knockdown of CRB3 by RNAi. The expression of OAS1 and STAT1 remained relatively stable, confirming specific CRB3 KD without apparent off-target effects. S16 served as a loading control. (**B**) A study by IB illustrated specific knockdown of CRB3 by RNAi up to ~50% did not perturb the steady-state levels of virtually all the BTB-associated proteins that were examined by immunoblotting, illustrating the lack of off-target effects, with the exception of formin 1 which was reduced by ~40% after CRB3 knockdown. Each bar in the histogram is a mean ± SD of 4 experiments. **P < 0.01 by Student’s *t*-test. (**C**) A study by IF to confirm CRB3 KD down-regulated its expression considerably. CRB3 KD also affected the localization of cell polarity protein PATJ, TJ proteins CAR and ZO-1, and basal ES proteins N-cadherin and β-catenin at the Sertoli cell-cell interface in which these proteins moved away from the cell junction site (see white brackets). In control, PATJ was localized at the cell-cell interface (white arrowheads), after CRB3 KD, it was internalized, and its localization at the cell-cell interface considerably diminished (yellow arrowheads). Sertoli cell nuclei were visualized by DAPI (blue). FI, fluorescence intensity; SC, Sertoli cells. siGLO transfection indicator (red) illustrated successful transfection. Scale bar, 30 μm. Histograms summarize IF results with each bar a mean ± SD of 3 experiments. **P < 0.01 by Student’s *t*-test. (**D**) CRB3 KD perturbed the Sertoli cell TJ-permeability barrier with each data point a mean ± SD of TER measurement of a representative experiment with triplicate bicameral units from *n* = 3 experiments that yielded similar results. *P < 0.05; **P < 0.01.

To probe the function of CRB3 in the testis, we used CRB3-specific siRNA duplexes *vs*. non-targeting negative control siRNA duplexes to KD CRB3 by RNAi in Sertoli cells as noted in Fig. 5A–D. CRB3 KD was shown to silence CRB3 mRNA expression by >70% (Fig. 5A) and a reduction in CRB3 protein expression by ~50% (Fig. 5B) without any apparent off-target effects since two interferon stimulated genes (ISG), including oligoadenylate synthetase 1 (OAS1) and signal transducer and activator of transcription 1 (STAT1), remained relatively steady (Fig. 5A), which were known to be up-regulated non-specifically when mammalian cells were transfected by siRNA duplexes with off-target effects^52^,^53^. Interestingly, CRB3 KD led to a significant down-regulation of formin 1 (Fig. 5B), a barbed end nucleation protein that is known to form long stretches of actin microfilaments in mammalian cells^54^,^55^, illustrating the possibility that CRB3 may play a role in regulating actin microfilament dynamics, consistent with data shown in Fig. 4 that CRB3 structurally interacts with Arp3 and Eps8. While CRB3 did not perturb the expression of BTB-associated proteins, its KD, as illustrated by considerably reduction in fluorescence intensity in Sertoli cells, induced changes in the distribution of its component protein PATJ, TJ proteins CAR and ZO-1, and basal ES proteins N-cadherin and β-catenin in which these no longer prominently (e.g., PATJ) or restrictively (e.g., TJ- and basal ES-proteins) expressed at the Sertoli cell-cell interface, but considerably diminished and internalized (Fig. 5C). These changes thus destabilized the Sertoli cell TJ-permeability barrier as noted in a study that monitored the TJ-barrier function following CRB3 KD (Fig. 5D).

### CRB3 regulates actin microfilament organization in Sertoli cells via its effects on Arp3, Eps8 and formin 1

**Figure 6:**
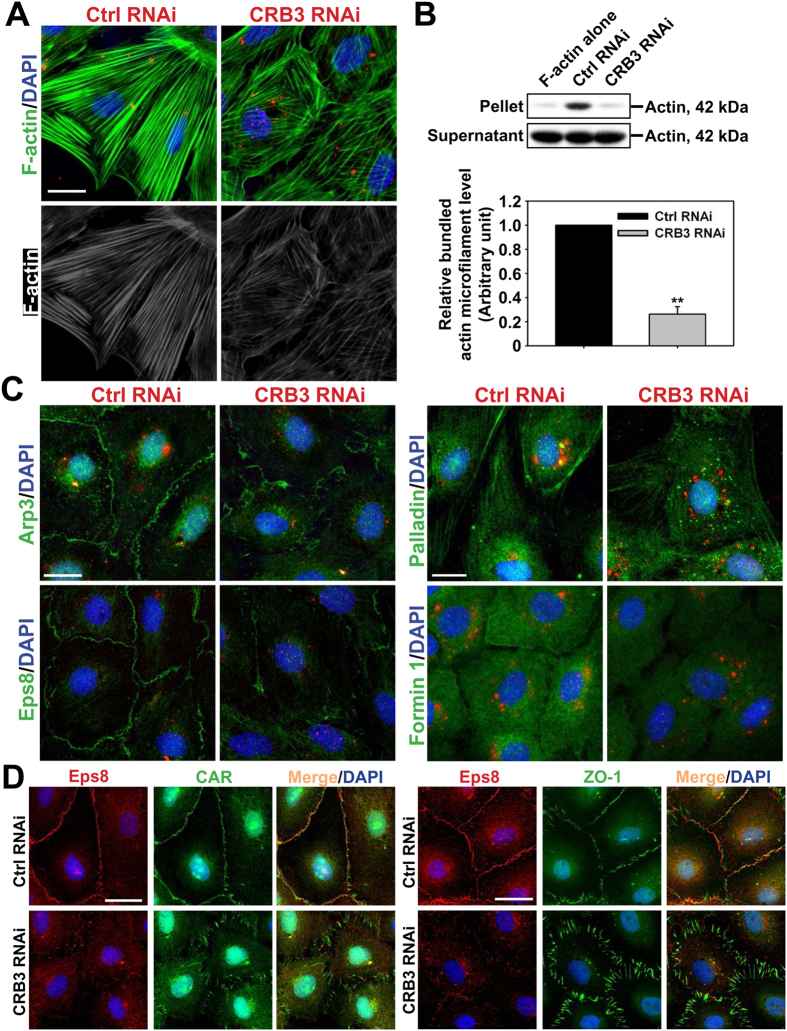
CRB3 knockdown causes disorganization of actin microfilaments in Sertoli cells mediated by changes in the spatiotemporal expression of actin binding and regulatory proteins. (**A**) After CRB3 KD, the F-actin (green or gray) network in Sertoli cells became truncated and branched, illustrating changes in their organization in which actin microfilaments no longer stretched across the entire Sertoli cell as noted in control cells, supporting the possibility of a reduced actin bundling capability. Scale bar, 20 μm. (**B**) A study by actin bundling assay supported the notion that CRB3 KD led to considerable reduction in actin bundling capability. In this biochemical assay, pellet contained bundled F-actin, whereas supernatant contained unbundled and linear as well as truncated actin microfilaments. Each bar in the histogram is a mean ± SD of *n* = 4 experiments. **P < 0.01 by Student’s *t*-test. (**C**) Changes in actin microfilament organization following CRB3 KD were shown to be mediated by changes in the spatiotemporal expression and/or localization of branched actin polymerization protein Arp3, which was internalized from near the cell surface, relocated to cell cytosol, thereby facilitating the organization of actin microfilaments into a branched network. Furthermore, actin barbed end capping and bundling protein Eps8 no longer localized to the cell-cell interface to maintain actin microfilament bundles to support the barrier function, instead, Eps8 became scattered across the cytosol. The actin cross-linking/bundling protein palladin distribution remained somewhat unaltered which continued to support the shorter actin microfilaments in Sertoli cells following CRB3 KD, whereas barbed end actin nucleation protein formin 1 was considerably down-regulated, consistent with immunoblotting data shown in Fig. 5B. Sertoli cell nuclei were visualized by DAPI (blue). siGLO transfection indicator (red) illustrated successful transfection. Scale bar, 20 μm. (**D**) To confirm re-distribution of actin regulatory proteins, such as Eps8, through its re-location from near the cell surface to cell cytosol, Eps8 before and after CRB3 knockdown was co-localized with a TJ-integral membrane protein CAR vs. a TJ adaptor protein ZO-1. Scale bar, 25 μm.

To better understand the molecular mechanism(s) underlying changes shown in Fig. 5 following CRB3 KD, we examined the organization of actin microfilaments in Sertoli cells after CRB3 silencing by RNAi since the basal ES/BTB integrity is contributed mostly by actin microfilament bundles^3^,^8^. When CRB3 in Sertoli cell epithelium with an established functional TJ-barrier was silenced by RNAi, actin microfilaments in Sertoli cells were shown to become truncated and branched, in contrast to control cells transfected with non-targeting negative control siRNA duplexes wherein actin microfilaments stretched across the Sertoli cell cytosol (Fig. 6A). A considerable and statistically significant reduction in the actin bundling capability in CRB3 KD Sertoli cells was noted based on a biochemical actin bundling assay (Fig. 6B). More important, this considerable loss of actin microfilament bundles in Sertoli cell cytosol was the result of an increase in branched actin polymerization-inducing protein Arp3 in cell cytosol, possibly via its internalization from the cell cortical zone, and a concomitant change in the distribution of actin barbed end capping and bundling protein Eps8, but not actin cross-linking and bundling protein palladin (Fig. 6C). Furthermore, there was a considerably reduction in barbed end nucleation protein formin 1 that is known to promote the generation of long stretches of actin microfilaments (Fig. 6C), consistent with the IB result shown in Fig. 5B. Findings shown in Fig. 6D further confirmed re-distribution of actin binding proteins (e.g., Eps8) following CRB3 knockdown. In control cells, Eps8 was co-localized with a TJ integral membrane protein CAR and a TJ adaptor protein ZO-1 at the Sertoli cell-cell interface, however, following CRB3 knockdown, the merged Eps8/CAR and Eps8/ZO-1 fluorescence staining appeared in orange fluorescence no longer found at the cell-cell interface but at the cell cytosol (Fig. 6D). Collectively, these findings illustrate that CRB3 may exert its effects via changes in the organization of actin microfilaments in Sertoli cells through the respective spatiotemporal localization of ABPs (e.g., Arp3, Eps8, formin 1) that modulate microfilament configuration at the basal ES/BTB. To further confirm the effects of CRB3 KD as reported herein were not off-target effects besides data shown in Fig. 5A, we had used two non-functional CRB3 siRNA duplexes of 21 nucleotides (RNAi #1 and RNAi #2) obtained from another vendor (see *Materials and Methods*) identified in our pilot experiments as internal controls. As noted in Figure S1, Sertoli cells transfected with these negative siRNA duplexes (i.e., CRB3 RNAi #1 and #2), successful transfection, illustrated by siGLO transfection indicator (red fluorescence), had no effects on the distribution of either CAR or Eps8, further confirming data shown in Fig. 6 were the results of CRB3 KD.

### CRB3 knockdown in the testis *in vivo* perturbs spermatid and phagosome transport during the epithelial cycle

**Figure 7:**
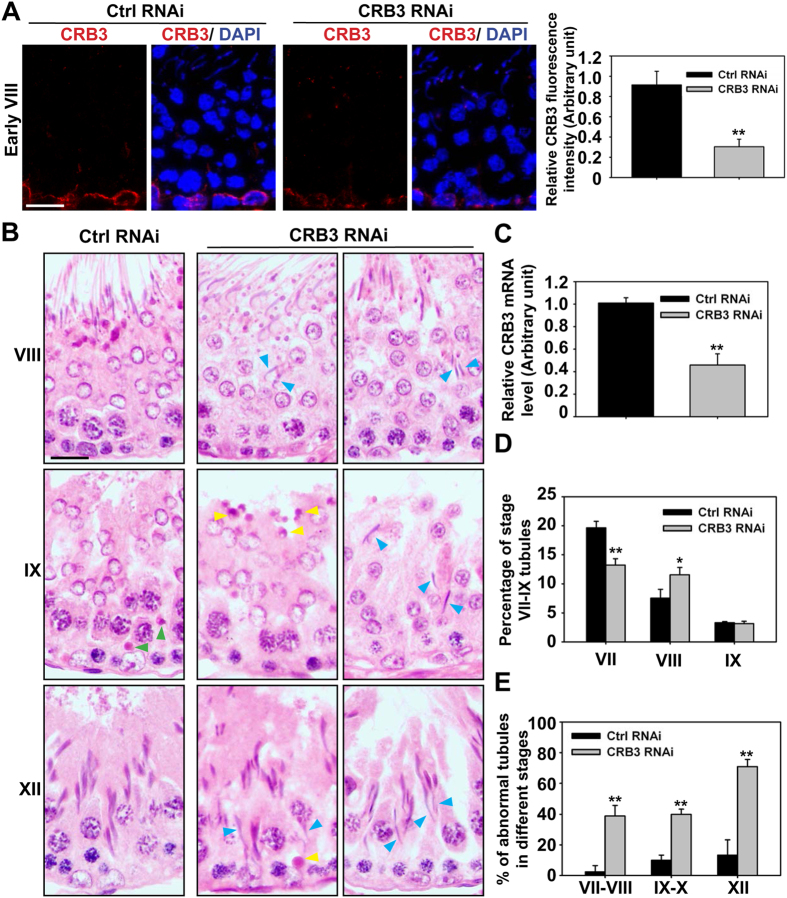
CRB3 knockdown in adult rat testes *in vivo* impairs spermatid and phagosome transport. Adult rat testes were transfected with corresponding siRNA duplexes on day 0. On day 5, testes were collected for IF or histological analysis. (**A**) Frozen sections of testes were stained for CRB3 (red), illustrating considerable CRB3 KD in this early stage VIII tubule. Cell nuclei were visualized by DAPI (blue). Scale bar, 30 μm. IF data were summarized in this histogram with each bar a mean ± SD of *n* = 3 rats using ~100 randomly selected tubules, illustrating a KD efficiency >70%. **P < 0.01. (**B**) In control RNAi testes, elongated spermatids lined up at the tubule lumen in this stage VIII tubule, ready for spermiation. However, elongated spermatids remained entrapped in the epithelium at stage VIII in the CRB3 RNAi group (blue arrowheads in the micrographs). In control testes, phagosomes were transported to the base of the epithelium at stage IX (green arrowheads) as earlier reported^59^. But CRB3 KD led to retention of phagosomes near the luminal edge (yellow arrowheads), illustrating defects in phagosome transport, with elongated spermatids remained entrapped in the epithelium in this early stage IX tubule (blue arrowheads). In Ctrl RNAi testes, only step 12 elongating spermatids were found in the epithelium of stage XII tubules; however, in CRB3 KD testes, step 19 spermatids were persisently found in stage XII tubules (blue arrowheads) and phagosomes also persisted in stage XII tubules (yellow arrowhead), illustrating CRB3 KD caused defects in spermatid and phagosome transport. Scale bar, 30 μm. (**C**) Steady-state mRNA level of CRB3 was estimated by qPCR, illustrating ~60% KD of CRB3. Each bar is a mean ± SD of *n* = 5 rats. **P < 0.01. (**D**) Percentage of stages VII and VIII tubules in the testes after CRB3 KD, but not IX tubules, was considerably altered after CRB3 KD. About 600 staged tubules from *n* = 3 rats were scored. *P < 0.05, **P < 0.01. E) Staged tubules that had defects in spermatid and/or phagosome transport as noted in (B) were scored with a total of ~300 tubules from *n* = 3 rats. **P < 0.01.

One of the major functions of the actin-based cytoskeleton, such as actin microfilament bundles at the apical and basal ES, is to support spermatid and phagosome transport^3^,^7^,^56^,^57^,^58^,^59^, we next expanded our observations *in vitro* to the testis *in vivo* by silencing CRB3 with high efficiency using the Polyplus *in vivo*-jetPEI as the transfection medium. CRB3 was knockdown by almost 70% in the testis in which fluorescence in the basal compartment was considerably diminished (Fig. 7A). Following CRB3 KD, step 19 spermatids were consistently found to be embedded deep inside the seminiferous epithelium in stage VIII, IX and even in XII tubules, see blue arrowheads in Fig. 7B, when CRB3 was silenced by ~60–70% in the testis *in vivo* based on analysis by IF (Fig. 7A) and qPCR (Fig. 7C). For instance, step 19 spermatids remained in the epithelium alongside with step 12 spermatids in stage XII tubules, see blue arrowheads in Fig. 7B. Furthermore, phagosomes remained near the luminal edge in stage IX tubules when they should have been transported to the base of the epithelium^59^,^60^, see yellow *vs.* green arrowheads in stage IX tubules in corresponding CRB3 KD and control testes in Fig. 7B. These defects in spermatid transport thus altered the appearance of stage VII and VIII tubules, changing the apparent % of VII and VIII tubules (Fig. 7D) wherein the frequency of stage VII and VIII tubules in control testes was ~20% and ~8%, respectively, consistent with findings of an earlier report in the rat testis^61^. In short, considerable defects in spermatid and phagosome transport in tubules noted in Fig. 7B were found to be statistically significant (Fig. 7E).

### CRB3 knockdown in the testis *in vivo* perturbs BTB protein distribution at the BTB due to changes in F-actin organization

**Figure 8:**
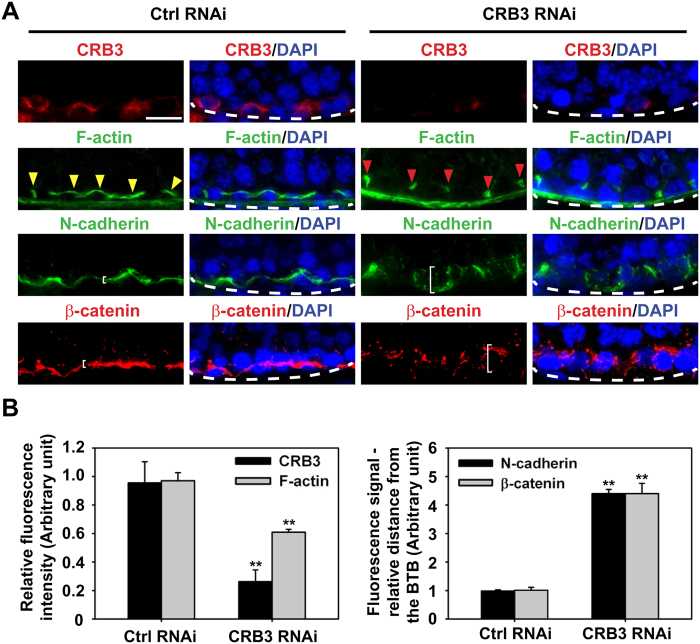
Changes in the distribution of F-actin, basal ES proteins N-cadherin and β-catenin at the BTB/basal ES after CRB3 knockdown in adult rat testes *in vivo*. Frozen sections obtained from testes transfected with CRB3 siRNA duplexes *vs.* non-targeting negative control duplexes were stained for CRB3, F-actin, basal ES proteins N-cadherin and β-catenin, which were localized at the basal ES/BTB. Cell nuclei were visualized by DAPI (blue). Stage VII tubules were randomly selected for analysis. (**A**) CRB3 KD was assessed by IF staining in which CRB3 (red) fluorescence intensity was considerably diminished. The fluorescence intensity of F-actin at the BTB was also considerably diminished following CRB3 KD in which F-actin no longer lined up properly along the BTB as found in control testes (see red arrowheads in CRB3 KD testes *vs.* yellow arrowheads in control testes). Furthermore, basal ES proteins N-cadherin and β-catenin were no longer tightly restricted to the BTB, but diffusely distributed at the site, consistent with findings shown in Fig. 5C (see white brackets in CRB3 RNAi group *vs*. Ctrl RNAi group). Basement membrane was annotated by dashed white line illustrating the relative location of the tunica propria. Scale bar, 30 μm. (**B**) Semi-quantitative analysis of fluorescence data shown in (A) including fluorescence intensity (left panel) *vs.* mis-localization of basal ES proteins by diffusing away from the BTB (right panel). Data in the control group were arbitrarily set at 1. Each bar is a mean ± SD of *n* = 3 rats. **P < 0.01.

CRB3 was silenced in the testis *in vivo* with an efficiency of ~70% (Fig. 8A,B), which also associated with a reduced fluorescence signal of F-actin, see green fluorescence in Fig. 8A such as in stage VII tubules when its expression should have been prominent^62^, illustrating reduced actin microfilaments at the basal ES/BTB (see red *vs.* yellow arrowheads in Fig. 8A) to support adhesion protein complexes at the BTB, consistent with findings of *in vitro* experiments shown in Figs 5 and 6. This notion was supported by observations wherein basal ES proteins N-cadherin and β-catenin no longer tightly localized at the BTB, but diffusing away from the site (Fig. 8A,B), also consistent with data shown in Fig. 5C.

### CRB3 knockdown in the testis *in vivo* perturbs apical ES protein distribution due to changes in F-actin organization

**Figure 9:**
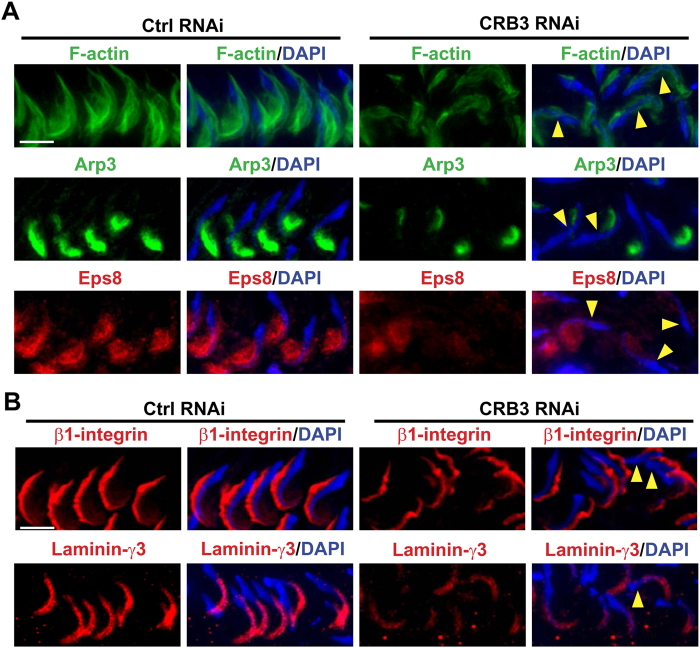
Changes in the distribution of F-actin, actin binding/regulatory proteins, and adhesion proteins at the apical ES following CRB3 knockdown in the testis *in vivo* that impede spermatid transport and polarity in the adluminal compartment. Frozen sections of testes following transfection with CRB3 siRNA duplexes *vs.* non-targeting negative control duplexes were fluorescently stained for F-actin (green), branched actin polymerization protein Arp3 (green), actin barbed end capping and bundling protein Eps8 (red) *vs.* adhesion proteins β1-integrin and laminin-γ3 at the apical ES. Cell nuclei were visualized by DAPI. (**A**) In stage VII tubules, F-actin was prominently localized to the concave (ventral) side of spermatid heads in control testes transfected with non-targeting negative control siRNA duplexes. However, CRB3 KD led to changes in actin organization in which F-actin no longer prominently localized to the concave side of spermatid heads but mostly to the convex (dorsal) side of spermatid heads. These changes thus caused defects in spermatid polarity in which many spermatid heads no longer pointing towards the basement membrane but deviated by at least 90° from the intended orientation (annotated by yellow arrowheads). These changes in F-actin organization were the result of changes in the spatiotemporal expression of Arp3 and Eps8 in which these actin regulatory proteins no longer restrictively expressed at the concave side of spermatid heads, but considerably diminished in particular in spermatids displaying defects in polarity (annotated by yellow arrowheads). Scale bar, 20 μm. (**B**) Adhesion proteins at the apical ES, such as β1-integrin (an integral membrane protein exclusively expressed by Sertoli cells at the apical ES) no longer tightly localized to the convex side of spermatid heads, but it was detaching from the site and its expression was considerably weakened, similar to the spatiotemporal expression of laminin-γ3 (a spermatid-specific apical ES adhesion protein) which prominently expressed at the spermatid head in control was considerably diminished after CRB3 KD. Scale bar, 20 μm.

CRB3 KD in the testis *in vivo* also impeded F-actin organization wherein F-actin no longer prominently expressed at the concave (ventral) side of spermatid heads as noted in control (Fig. 9A). Such changes in F-actin organization at the apical ES was likely the result of changes in the spatiotemporal expression of branched actin polymerization protein Arp3 and actin barbed end capping and bundling protein Eps8 (Fig. 9A), rendering F-actin network to become disorganized at the site. Since adhesion protein complexes, such as ß1-integrin-laminin-γ3^63^,^64^ at the apical ES utilized F-actin for attachment, when F-actin organization at the apical ES was impeded (Fig. 9A), spermatid adhesion was thus disrupted, perturbing spermatid polarity as earlier reported^28^. Indeed, considerable changes in the spatiotemporal expression of β1-integrin (a Sertoli cell-specific apical ES protein) and laminin-γ3 (a spermatid-specific apical ES protein) was detected (Fig. 9B). In short, changes in the spatiotemporal expression of Arp3 and Eps8 that contributed to an alteration of F-actin organization thus impeded apical ES adhesion protein complex function, leading to defects in spermatid polarity, in which spermatid heads were deviated by at least 90° (annotated by yellow arrowheads) from the intended orientation of pointing toward the basement membrane in many spermatids shown in Fig. 9A,B. Furthermore, changes in spatiotemporal expression of Arp3 and Eps8 (Fig. 9) also disrupted the plasticity of apical ES to convert actin microfilaments efficiently between their bundled and unbundled/branched configuration to support cell/organelle transport during the epithelial cycle, which is one of the most crucial functions of the apical ES (for a review, see ref. 2), leading to defects in spermatid and phagosome transport shown in Fig. 7.

## Discussion

CRB3, a small integral membrane protein of 24 kDa in mammalian cells including the Sertoli cell, creates a 3-protein polarity complex with PALS1 and PATJ (for reviews, see refs 18, 19 and 65). The CRB3/PALS1/PATJ protein complex, together with the Par3/Par6/aPKC (atypical protein kinase C) and the Scribble/Lgl (Lethal giant larvae)/Dlg (Discs large) polarity complexes are the three known polarity complex modules that regulate the formation of apico-basal polarity during embryonic development, maintain cell polarity and adhesion, and the assembly and maintenance of TJ, adherens junction (AJ) in adult tissues in virtually all organs including the testis (for reviews, see refs 17, 18, 19 and 66). Recent studies have shown that components of these three polarity complex modules that confer cell polarity, and modulate the assembly and maintenance of cell junctions including adhesion, in particular during tumorigenesis are mediated through F-actin-based cytoskeleton (for reviews, see refs 67, 68, 69). This is not entirely unexpected since some of the component proteins in these polarity proteins, such as PALS1, PATJ in the Crumbs-, Par6 and aPKC in the Par-, and Lgl and Dlg in the Scribble-based protein complex module can recruit other regulatory proteins to create a giant protein complex to regulate actin microfilament functions. For instance, Cdc42, a member of the Rho GTPases and a known regulator of actin- and microtubule-based cytoskeletal function (for reviews, see refs 70 and 71) via its effects on endocytic vesicle-mediated protein trafficking (for a review, see ref. 72), is an integrated component of the Par-based polarity complex (for reviews, see refs 19 and 49), has been shown to be involved in TGF-β3-mediated Sertoli cell BTB disruption via an increase in endocytosis of integral membrane proteins at the Sertoli cell BTB, such as occludin and CAR (coxsackievirus and adenovirus receptor, a TJ integral membrane protein)^73^. Specifically, TGF-β3-induced BTB disruption requires an activation of Cdc42 to its active GTP-bound form, and overexpression of a dominant negative Cdc42 mutant that inactivates Cdc42 function in Sertoli cells blocks the TGF-β3 disruptive effects on the TJ-barrier function^73^. Since this TGF-β3/Cdc42-mediated vesicle trafficking event is dependent on F-actin near the Sertoli cell plasma membrane, these findings provide the compelling evidence that the Par-based polarity complex exerts its effects through the actin microfilaments at the basal ES/BTB. Furthermore, a knockdown (KD) of Scribble, Lgl2 and Dlg1 at the Sertoli cell BTB using an *in vitro* model, as well as a KD of Scribble/Lgl2/Dlg1 in the testis *in vivo* was found to promote the TJ-permeability barrier by recruiting more TJ-protein occludin and/or basal ES protein β-catenin at the Sertoli cell-cell interface to tighten the TJ^43^. Herein, the KD of CRB3 in Sertoli cells with an established TJ-barrier was shown to perturb the TJ-permeability by causing truncation of the actin microfilaments, also, these actin filaments were found to be branched, likely the result of changes in the spatiotemporal expression of branched actin inducing protein Arp3 and actin barbed end capping and bundling protein Eps8, as well as a concomitant down regulation of barbed end nucleation protein formin 1. These findings thus illustrate the contrasting action of CRB3- *vs.* Scribble-based polarity complex in which CRB3 promotes whereas Scribble perturbs^43^ the Sertoli cell TJ-barrier function in the normal testis. This is not entirely unexpected since it is known that the localization of the CRB3- (which usually is working in concert with the Par-based complex at the TJ) and Scribble-based protein complex is mutually exclusive (for reviews, see refs 19 and 49), which is necessary to maintain apico-basal polarity in a cell epithelium. As such, they can also have contrasting functionality in modulating the F-actin network at the Sertoli cell BTB. The notion that CRB3 exerts its effects through actin-based cytoskeleton is consistent with the findings that CRB3 structurally interacts with Eps8 and Arp3 in the rat testis, which are known to have contrasting effects on the actin microfilament bundles in which Eps8 promotes actin microfilaments to organize into bundles whereas Arp3 promotes an unbundled/branched configuration^51^,^74^. A recent report in *Drosophila* has suggested that CRB regulates cytoskeletal dynamics and cell adhesion during dorsal closure in the brain and this function of CRB requires the involvement of Arp2/3^75^, supporting the notion that CRB3 is likely working in concert with actin binding protein Arp3 to modulate cytoskeletal function. Also, findings that used CRB3 KO mice also support the notion that CRB3 is involved in actin-based cytoskeleton. For instance, a deletion of CRB3 in mice leads to intestinal abnormalities in which microvilli are extremely shortened; also the brush borders (which are also microvillus-based structures) in proximal tubules of the kidney are severely shortened, displaying partial loss, possibly due to a defect in the formation of membrane cytoskeleton^27^. Furthermore, this phenotype mimics the defects found in the intestinal epithelial cells and microvilli of the *Erz* (ezrin) KO mice^76^,^77^, and ezrin is recently shown to be an important actin regulator by inducing bundling actin microfilaments at the ES in rat testes^78^. Collectively, these findings thus support the notion that CRB3 is likely working in concert with Eps8, Arp3 and possibly ezrin to modulate actin microfilaments at the basal ES/BTB.

Findings obtained in studies *in vitro* using a Sertoli cell model that mimics the BTB *in vivo* have been confirmed in experiments *in vivo* when CRB3 was KD in the testis using the *in vivo*-jetPEI as the transfection medium with high efficiency as recently reported^79^,^80^. Based on the analysis reported herein, CRB3 KD in the testis *in vivo* led to at least a ~60% silencing of CRB3 expression, matching the efficacy based on RNAi results *in vitro*. CRB3 KD was found to perturb the F-actin network at the basal and apical ES in the testis, leading to defects in spermatid and phagosome transport, which are known F-actin-dependent events (for reviews, see refs 2, 59 and 60). For instance, step 19 spermatids were persistently detected in stage IX through XII tubules, deeply embedded into the seminiferous epithelium, whereas phagosomes that should have been transported to the basal compartment at stage IX-X for their degradation (for a review, see ref. 59) remained at the luminal edge of the epithelium, long after spermiation has taken place, and some phagosomes were detected in stage XII tubules. These findings are not entirely unexpected, since it is noted that the transport of organelles (e.g., phagosomes) and spermatids rely on the networks of polarized actin microfilaments and microtubules (for reviews, see refs 2, 81 and 82).

In this context, it is of interest to note that while CRB3 is a small transmembrane protein, yet it is also localized prominently in the Sertoli cell cytosol, consistent with findings in other mammalian cells^41^,^45^. Studies in occludin, a TJ-integral membrane protein, have shown that while it is localized at the TJ-fibrils at the cell-cell interface^83^, which is phosphorylated at the Ser/Thr and Thr residues^84^,^85^, such as in Sertoli cells^86^,^87^. However, a minor pool of occludin is detected in cell cytosol near the basolateral region, which is less phosphorylated and not assembled into TJ fibrils^85^,^88^. This lateral pool of occludin likely serves as a reserve of occludin molecules for rapid expansion of TJs. Thus, CRB3 likely serves as a regulatory protein besides a structural component of the Crumbs-based polarity complex that modulates rapid expansion or degeneration of ES in response to changes of the epithelial cycle.

In summary, CRB3 is a novel regulator of actin microfilaments at the ES in adult rat testes through its effects on the actin binding and regulatory proteins Arp3 and Eps8, modulating the organization of actin filaments.

## Additional Information

**How to cite this article**: Gao, Y. *et al.* Polarity protein Crumbs homolog-3 (CRB3) regulates ectoplasmic specialization dynamics through its action on F-actin organization in Sertoli cells. *Sci. Rep.* **6**, 28589; doi: 10.1038/srep28589 (2016).

## Supplementary Material

Supplementary Information

## Figures and Tables

**Table 1:** Antibodies used for different experiments in this report.

				Working dilution	
Antibody	Host species	Vendor	Catalog no.	IB/IP	IF/IHC
Actin	Goat	Santa Cruz Biotechnology	sc-1616	1:300	
aPKC	Rabbit	Santa Cruz Biotechnology	sc-216	1:1000	
Arp3	Mouse	Sigma-Aldrich	A5979	1:3000/2 μg	1:50
α-tubulin	Mouse	Abcam	ab7291	1:1000	
β-catenin	Rabbit	Invitrogen, Life Technologies	71-2700	1:250	1:100
CAR	Rabbit	Santa Cruz Biotechnology	sc-15405	1:200	1:50
Cdc42	Mouse	Millipore	05–542	1:250	
Claudin-11	Rabbit	Invitrogen, Life Technologies	36–4500		1:100
CRB3	Rabbit	Margolis Lab^41^			1:250
CRB3	Rabbit	Sigma-Aldrich	HPA013835	1:1000	1:100/1:50
CRB3	Rabbit	Abcam	ab173169	1:500	
CRB3	Rabbit	Santa Cruz Biotechnology	sc-292449	2 μg	
Eps8	Mouse	BD Biosciences	610143	1:5000/2 μg	1:50
Formin 1	Mouse	Abcam	ab68058	1:500	1:50
GAPDH	Mouse	Abcam	ab8245	1:1000	
β1-Integrin	Rabbit	Millipore	AB1952		1:100
JAM-A	Rabbit	Invitrogen, Life Technologies	36–1700	1:250	
Laminin-γ3	Rabbit	Cheng Lab^63^			1:300
N-cadherin	Mouse	Invitrogen, Life Technologies	33–3900	1:200	1:100
Occludin	Rabbit	Invitrogen, Life Technologies	71–1500	1:250	
Occludin	Mouse	Invitrogen, Life Technologies	33–1500		1:100
Palladin	Rabbit	Proteintech	10853-1-AP	1:1000/2 μg	1:100
PALS1	Rabbit	Millipore	07–708	1:1000	
Par3	Rabbit	Millipore	07–330	1:500	
Par6	Rabbit	Abcam	ab45394	1:1000	
PATJ	Rabbit	Abcam	ab102113		1:50
PATJ	Goat	Abcam	ab8225	1:500	
Plastin 3	Rabbit	Abcam	ab137585	1:500	
Scribble	Goat	Santa Cruz Biotechnology	sc-11048	1:500	
Vimentin	Mouse	Santa Cruz Biotechnology	sc-6260	2 μg	
ZO-1	Rabbit	Invitrogen, Life Technologies	61–7300	1:250	1:100
ZO-1	Mouse	Invitrogen, Life Technologies	33–9111		1:100

Abcam, Cambridge, MA; Santa Cruz Biotechnology, Santa Cruz, CA; Sigma-Aldrich, St. Louis, MO; Invitrogen, Life Technologies, Carlsbad, CA; Proteintech, Chicago, IL; BD Biosciences, San Jose, CA; Millipore Corp., Billerica, MA.

**Table 2:** Primers used for RT-PCR and qPCR experiments in this report.

Gene	Primer Sequence (5′ to 3′)	Position	Length (bp)	Tm (°C)	Number of cycle	GenBank accession number
CRB3	Sense: GGCGTTTGGCTTGCCGAT	30–47	245	61	30	NM_001025661.1
	Antisense: CACTGCTGGGCCGGTAG	258–274				
GAPDH	Sense: GCTGGTCATCAACGGGAAAC	192–211	112			NM_017008.4
	Antisense: GGTGAAGACGCCAGTAGAC-	285–303				
OAS1A	Sense: GAGTGAAGTTTGAGGTCCAGA	350–370	230	54.5	30	NM_138913
	Antisense: CTCCGTGAAGCAGGTAGA	562–579				
STAT1	Sense: GAGTGGAAGCGAAGACAG	712–729	218	54.5	30	NM_032612
	Antisense: TGGAAGAGGACGAAGGTG	912–929				
S16	Sense: TCCGCTGCAGTCCGTTCAAGTCTT	15–38	385			XM_341815
	Antisense: CCAAACTTCTTGGTTTCGCAGCG	376–399				
