# Supplementary material for: Polarity protein Crumbs homolog-3 (CRB3) regulates ectoplasmic specialization dynamics through its action on F-actin organization in Sertoli cells
Source: Sci Rep. 2016 Jun 30;6:28589. doi: 10.1038/srep28589 (PMC4928075; doi:10.1038/srep28589)

Supplementary Information

Polarity protein Crumbs homolog-3 (CRB3) regulates ectoplasmic specialization dynamics through its action on F-actin organization in Sertoli cells

Ying Gao,[1] Wing-yee Lui,[2] Will M. Lee,[2] C. Yan Cheng[1]

Figure S1. Transfection of Sertoli cell epithelium with two non-functional CRB3 siRNA duplexes had no effects on steady-state level of CRB3 and localization of CAR and Eps8. Sertoli cells were transfected with CRB3 RNAi #1 and #2, two non-functional CRB3 siRNA duplexes identified in our pilot experiments (see *Materials and Methods*) and detailed in *Materials and Methods* (see also legend to **Figure 6**) *versus* the non-targeting negative control siRNA duplexes (Ctrl RNAi). In brief, 150 nM siRNA duplexes were used for double transfections for immunoblotting experiments, and 100 nM siRNA duplexes were used for immunofluorescence analysis (single transfection). Successful transfection was confirmed by siGLO indicator (red fluorescence). These transfections failed to induce changes in the steady-state level of CRB3 in Sertoli cells as illustrated by immunoblotting. They also failed to affect the distribution of CAR (a TJ-protein) and Eps8 at the Sertoli cell-cell interface, unlike the knockdown of CRB3 in Sertoli cells as noted in **Figure 6**. Scale bar, 20 µm, which applies to other micrographs in this panel.

Figure S1 (Gao et al.)

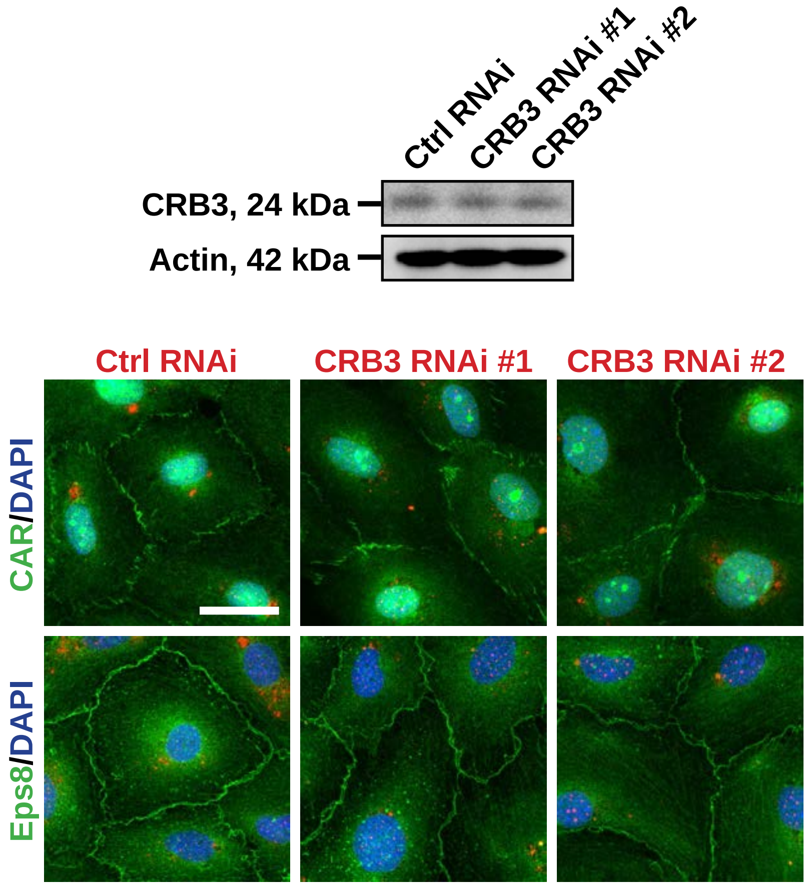

Supplement: Supplementary Information [file srep28589-s1.pdf]
